# Systemic treatment approaches in traumatized refugees: data on service use from Bologna prospective

**DOI:** 10.1192/j.eurpsy.2026.10338

**Published:** 2026-07-31

**Authors:** S. Giudice, I. Tarricone, F. Lucchi

**Affiliations:** 1 Department of Mental Health and Pathological Addictions, AUSL DI BOLOGNA; 2 Department of Mental Health and Pathological Addictions, Department of Medical and Surgical Sciences, Alma Mater Studiorum - Bologna University, BOLOGNA, Italy

## Abstract

**Background:**

Refugees and Migrants exposed to war, persecution and forced displacement present complex menthal health needs, frequently characaterized by trauma related disorders and social vulnerability.Systemic and network-oriented interventions have been proposed as effective strategies to address such complexity,yet empirical data on their impact on menthal health.

**Objectives:**

to describe menthal health service utilization and continuity of care among traumatized refugees receinving systemic oriented treatment within a public menthal health setting in Bologna-Italy.

**Methods:**

this prospective observational study was conducted in a specialized outpatient service for refugees and migrants integrated into the local public menthal health system. Adult refugess or migrants with a history of traumatic exposure and clinically significant psychological distress were consecutively enrolled and followed for 12 mounths (2025). Data on sociodemographic characteristics, psychiatric diagnoses, type of interventions, outpatient contacts, emergency service use and psychiatric hospitalizations werw systematically collected.Systemic treatment approches included family and network-oriented intervetions, multidisciplinary case management and structured collaboration with social and community services.

**Results:**

During 2025, 147 Out of 147 patients, 103 (70%) presented with mental health issues. The cohort was predominantly male (54%), with the most frequent countries of origin being Pakistan, Tunisia, and Nigeria. A high prevalence of Post-Traumatic Stress Disorder (PTSD) was observed, frequently in comorbidity with other psychiatric disorders. Patients with PTSD received trauma-oriented psychotherapy, including EMDR, Narrative Exposure Therapy (NET), and bottom-up approaches. Notably, systemic interventions were provided to 68% of the total sample and were significantly associated with higher .

**Conclusions:**

Within a public mental health framework, systemic treatment approaches appear to promote engagement and continuity of care among traumatized refugees, resulting in relatively low rates of acute service use. These findings support the implementation of integrated, network-oriented models to address the clinical and social complexity of refugee mental health, optimizing resource allocation and patient outcomes rates of continuity of care.

**Disclosure of Interest:**

None Declared

